# Intraperitoneal hepato-renal toxicity of zinc oxide and nickel oxide nanoparticles in male rats: biochemical, hematological and histopathological studies

**DOI:** 10.17179/excli2023-6237

**Published:** 2023-07-09

**Authors:** Noura S. Abouzeinab, Nour Kahil, Najla Fakhruddin, Ramadan Awad, Mahmoud I. Khalil

**Affiliations:** 1 Department of Biological Sciences, Faculty of Science, Beirut Arab University, Beirut, Lebanon; 2 Department of Pathology and Laboratory Medicine, Cleveland Clinic Foundation, Cleveland, OH, USA; 3 Department of Physics, Faculty of Science, Beirut Arab University, Beirut, Lebanon; 4 Department of Physics, Faculty of Science, Alexandria University, Alexandria, Egypt; 5 Molecular Biology Unit, Department of Zoology, Faculty of Science, Alexandria University, Alexandria, Egypt

**Keywords:** nanoparticles, zinc oxide, nickel oxide, hepatotoxicity, nephrotoxicity, intraperitoneal route

## Abstract

In recent years, zinc oxide (ZnO) and nickel oxide (NiO) nanoparticles (NPs) have become more prevalent in commercial and industrial products. However, questions have been raised regarding their potential harm to human health. Limited studies have been conducted on their intraperitoneal toxicity in rats, and their co-exposure effects remain uncertain. Therefore, this study aimed to investigate some biological responses induced by a single intraperitoneal injection of ZnO-NPs (200 mg/kg) and/or NiO-NPs (50 mg/kg) in rats over time intervals. Blood and organ samples were collected from 36 male rats for hematological, biochemical, oxidative stress, and histological analysis. Results showed that the administration of NPs reduced the body and organ weights as well as red blood cell (RBC) indices and altered white blood cell (WBC) and platelet (PLT) counts. The experimental groups exhibited elevated levels of aspartate aminotransferase (AST), alanine transaminase (ALT), creatinine (CREA), urea, lipid profile, glucose (GLU), total protein (TP), albumin (ALB) and malondialdehyde (MDA), and decreased uric acid (UA), superoxide dismutase (SOD), and glutathione (GSH). Histological observations also revealed architectural damages in liver and kidneys. These alterations were time-dependent and varied in their degree of toxicity. Co-exposure of NPs initially lessened the damage but increased it afterwards compared to individual exposure. In conclusion, intraperitoneal injection of ZnO-NPs and/or NiO-NPs alters biological processes and induces oxidative stress in rats' liver and kidneys in a time-dependent manner, with NiO-NPs being more potent than ZnO-NPs. Furthermore, co-exposed NPs initially appeared to be antagonistic to one another while further aiming toward synergism.

## Introduction

Nanoparticles (NPs) are materials that exhibit unique physiochemical characteristics that differ from their bulk forms owing to their size ranging between 1 and 100 nm (Khan et al., 2019[38]). They are involved in a wide range of applications, such as paints, chemical sensors, advanced batteries, cosmetics, food, and food packaging (Sriva Prasanna et al., 2019[62]). Consequently, the continuing exposure to NPs has raised concerns about their potential adverse effects on human health and the environment (Pourmand and Abdollahi, 2012[53]). Having a small size, they have the ability to easily enter the human body, traverse different biological barriers, and reach the most vulnerable organs including the liver, kidney, and lungs (Ahamed et al., 2010[5]; Pourmand and Abdollahi, 2012[53]). Among NPs, metal oxide NPs (MNPs) have attracted greater attention, due to their outstanding features (Negrescu et al., 2022[48]). A number of metal oxide NPs have been synthesized and applied in different fields, such as titanium dioxide (TiO_2_), silicon dioxide (SiO_2_), iron oxide (FeO), zinc oxide (ZnO), copper oxide (CuO) and nickel oxide (NiO) (Sriva Prasanna et al., 2019[62]). 

Zinc oxide nanoparticles (ZnO-NPs) have found diverse applications in agriculture, medicine, food packaging, cosmetics, and various other fields (Rasmussen et al., 2010[55]). Despite being listed as a "generally recognized as safe" (GRAS) substance by the U.S. Food and Drug Administration, high concentrations of ZnO-NPs have been found to be toxic, while the long-term safety of low concentrations remains uncertain (Chang et al., 2012[22]; Abbasi et al., 2019[2]). Early investigations have identified the liver and kidneys as primary target organs for ZnO-NPs accumulation (Yan et al., 2012[70]; Chen et al., 2016[23]). ZnO-NPs mainly generate oxidative stress and suppress antioxidant mechanisms in liver and kidney cells (Xiao et al., 2016[69]; Yao et al., 2019[71]). Furthermore, ZnO-NPs have been reported to cause mitochondrial and DNA damage, and activate different cell death pathways (Yan et al., 2012[70]; Yao et al., 2019[71]; Almansour et al., 2019[14]). Moreover, ZnO-NPs can induce inflammation in liver and kidneys (Tang et al., 2016[65]; Almansour et al., 2019[14]).

Nickel oxide nanoparticles (NiO-NPs) are extensively studied due to their electrocatalysis, high chemical stability, conductance properties, and ability to transfer electrons (Thema et al., 2016[66]). These properties make them useful in a variety of fields, including biomedicine, where their antibacterial and anti-inflammatory properties have been recognized (Din and Rani, 2016[24]). However, studies have also shown that NiO-NPs can be toxic to animals and microorganisms such as algae, bacteria, and plants (Cameron et al., 2011[20]). The International Agency for Research on Cancer (IARC) has classified nickel and all nickel compounds as carcinogenic, further increasing concerns about their potential hazards (IARC, 1990[35]). NiO-NPs have been found to accumulate in cells, tissues, and organs, interacting with biomolecules and affecting their proteomic and metabolomic profiles, potentially inducing genotoxicity and carcinogenic effects (Dumala et al., 2018[25]). In rats, NiO-NPs have been found to induce hepatotoxicity by increasing reactive oxygen species (ROS) production, apoptosis in human liver cells (HepG2), and inflammation in the liver (Magaye et al., 2014[42]; Ahmad et al., 2015[6]). Additionally, high concentrations of metallothionein in the kidneys may lead to Ni accumulation and subsequent nephrotoxicity (Novelli et al., 1998[49]).

ZnO-NPs and NiO-NPs must undergo sufficient testing in order to establish a conclusion concerning their toxicity in biological systems, before their use becomes more prevalent in daily lifestyle. Despite previous reports on the toxicity of these NPs, there is a lack of information on their combined effects. As ZnO-NPs and NiO-NPs can coexist in real-life settings, it is critical to examine the adverse outcomes that may result from their combination, particularly since previous research has focused primarily on their individual toxic effects. Moreover, little attention has been devoted to the biological interaction pattern that occurs following their intraperitoneal (*i.p.*) administration in rats. Therefore, this study was conducted to assess the *in vivo* toxicity of ZnO-NPs and/or NiO-NPs in rats by investigating hematological, biochemical, and histological analyses, with a focus on their hepatotoxic and nephrotoxic effects. 

## Materials and Methods

### Nanoparticles

ZnO-NPs and NiO-NPs were synthesized by the co-precipitation method. These NPs were then characterized by x-ray powder diffraction (XRD) and transmission electron microscope (TEM) in the Department of Physics, Beirut Arab University. According to the results obtained by the researchers, ZnO-NPs displayed a distorted hexagonal shape, with a size equal to 68.14 nm and a surface area of 8.56x10^3^ (Kamareddine et al., 2020[36]). NiO-NPs were spherical in shape with a size of 28.87 nm and a surface area equal to 5.98x10^4^ (Al Boukhari et al., 2020[8]). 

### Animal husbandry and welfare

36 healthy male Sprague Dawley rats (*Rattus norvegicus*), weighing 150-250 g (aged 6 to 7 weeks old) were obtained from the animal house division of the Faculty of Science, Beirut Arab University. Rats were housed in large polypropylene stainless-steel cages provided with wood shaving, standard pellet diet, and ad libitum water. Animals were kept in a well-ventilated room at a constant temperature 23±2 ⁰C and relative humidity of 60-70 % with an artificial regimen of 12h light/dark cycle, at least one week prior to the experiment, for acclimatization. Animals handling, management, and experimental design and procedure were approved by the ethics committee “Institutional Review Board” (IRB), of Beirut Arab University, Lebanon (IRB protocol number: 2023-A-0049-S-M-0512). All laboratory animals received humanely care in compliance with the guidelines of the National Institutes of Health (NIH).

### Experimental design and dosage

**Figure 1:**
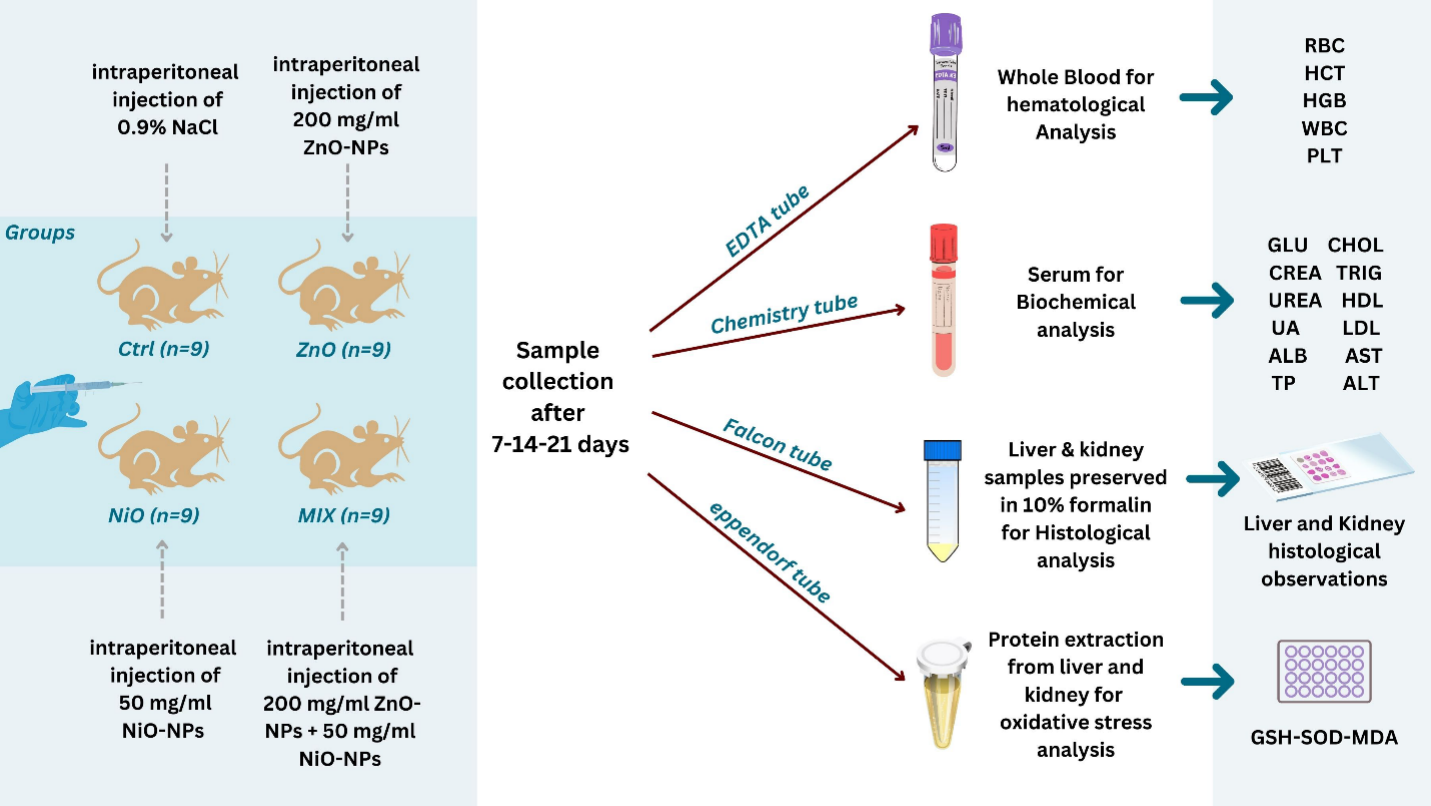
Schematic diagram of the experimental design for the current in vivo study. These steps included i.p. injection of NPs in four groups of rats (Ctrl, ZnO, NiO and MIX). Samples were collected on days 7, 14 and 21 following sacrifices; blood was collected in two different tubes, EDTA tube (prevent clotting) for whole blood analysis and chemistry tube (allows clotting) for biochemical analysis. Liver and kidneys were divided into two parts, one part was preserved in 10 % formalin for histological analysis and the other part was stored in -80 ⁰C for protein extraction to determine oxidative stress biomarkers.

ZnO-NPs and NiO-NPs were prepared using a sonication method in 0.9 % NaCl solution as a solvent. Doses of both NPs were determined initially from a pilot study conducted prior to the start of the experiment and showed hazardous effects in male rats without mortality. An extra group of 30 healthy adult male S.D. rats were exposed to different doses of ZnO-NPs and NiO-NPs, depending on the doses chosen in previous studies (Amara et al., 2014[15]; Abbasalipourkabir et al., 2015[1]; Katsnelson et al., 2015[37]; Marzban et al., 2020[43]; Sudhakaran et al., 2020[63]). Based on the pilot study and dose-response data, 200 mg/kg for ZnO-NPs and 50 mg/kg for NiO-NPs were selected, indicating that the LD_50_ for both NPs is higher than these doses. Prior to treatment, suspensions were vigorously stirred using a vortex mixer. 36 Sprague-Dawley rats were randomly divided into 4 sets, each set subdivided into 3 subgroups of 3 rats, and sacrificed on the 7^th^, 14^th^, and 21^st^ day of exposure. Control rats received a single i.p. injection of 0.9 % NaCl solution. The ZnO and NiO groups received single i.p. injections of ZnO-NPs (200 mg/kg b.w.) and NiO-NPs (50 mg/kg b.w.), respectively. The MIX group received both injections of ZnO-NPs (200 mg/kg b.w.) and NiO-NPs (50 mg/kg b.w.) separately via i.p. route to avoid direct interactions between the two NPs and to ensure that the exact volume of each is drawn. Rats were monitored daily for clinical signs of toxicity, and their body weight was measured twice a week throughout the experiment. Organ weight and indices were recorded upon sacrifice. The schematic diagram of the experimental design is shown in Figure 1(Fig. 1). All the experiments were carried out in triplicates and the results are reproduced in three independent experiments.

### Hematological and biochemical parameters

Following euthanization, blood was withdrawn from each rat by cardiac puncture. Various hematological parameters including red blood cell count (RBC), hemoglobin (Hb) content, Hematocrit (HCT), as well as platelets (PLT) and white blood cell (WBC) counts were analyzed using automated blood analyzer (Sysmex Xn-350, Germany). Serum biochemical parameters, included serum aspartate aminotransferase (AST), alanine transaminase (ALT), creatinine (CREA), urea, uric acid (UA), cholesterol (CHOL), triglyceride (TG), high-low density lipoprotein (HDL & LDL), glucose (GLU), albumin (ALB) as well as total protein (TP), and were determined using commercially available diagnostic kits (Spinreact, Girona, Spain), and measured spectrophotometrically (Gilford, Ciba-Corning Diagnostics, Oberlin, Ohio, USA). 

### Oxidative stress markers

Parts of liver and kidney tissue samples were homogenized with 0.1 M sodium phosphate buffer (PH 7.4) containing a protease inhibitor cocktail III. Following centrifugation of the homogenate at 10,000 rpm for 15 min, the supernatant was used for the following antioxidant assays: superoxide dismutase SOD, reduced glutathione GSH, and malondialdehyde MDA. 

### Superoxide dismutase (SOD)

Superoxide dismutase (SOD) was measured in tissues using the method described by Giannopolitis & Ries (1997[28]). It involves a competitive inhibition of nitro blue tetrazolium (NBT) by SOD enzymes. One unit of SOD activity is referred as the amount of protein that results in a 50 % inhibition of NBT reduction, which is measured spectrophotometrically at 560 nm using 96 well microplate reader (Multiskan™ FC Thermo Fisher Scientific Inc., USA).

### Reduced glutathione (GSH)

Glutathione (GSH) was measured in tissues using the method described by Moron et al. (1979[46]). It involves spectrophotometric detection of a yellow-colored product formed from the reaction between GSH and 5,5′-dithiobis-2-nitrobenzoic acid (DTNB). The reduced chromogen is directly proportional to the GSH concentration and its absorbance was measured at 405 nm using 96 well microplate reader (Multiskan™ FC Thermo Fisher Scientific Inc., USA).

### Malondialdehyde (MDA)

Lipid peroxidation was assayed by measuring the level of MDA according to the method described by Heath & Packer (1968[32]). This method specifically measures malondialdehyde (MDA); an end product of lipid peroxidation that interacts with thiobarbituric acid (TBA) to produce a pink species absorbed spectrophotometrically at 535 nm as a measure of lipid peroxidation using 96 well microplate reader (Multiskan™ FC Thermo Fisher Scientific Inc., USA).

### Histopathology and gross pathology

Euthanized animals were macroscopically examined for visible alterations and underwent histopathological analysis of liver and kidney tissues. Parts from liver and kidney tissues were fixed with 10 % formalin, embedded with paraffin and sectioned with rotary microtome (Leica RM2235, Leica Microsystems, USA). The sections were then stained with Harris hematoxylin and Eosin (H&E) and examined under light microscope (Leica DM500 with ICC50 W CAM, Leica Microsystems, USA). The average injury score was calculated based on several criteria including: damaged hepatocytes, cytoplasmic vacuolization, inflammatory cell infiltration, Kupffer cells hyperplasia, hepatocyte necrosis, pyknosis, and bile duct proliferation in liver; whereas glomerular atrophy, tubular degeneration and vacuolization, brush border loss, and necrotic tubules in kidneys. The degree of injury to liver and kidneys were determined semi-quantitatively using a 5-point scale from 0 to 5 as follows: 0= normal, 1= mild, 2= moderate, 3= moderate to severe, 4= severe, 5= highly severe, by applying the generic criteria described by previous researchers (Park et al., 2008[51]; Schafer et al., 2018[57]). 

### Statistical analysis

The values were expressed as mean ± SEM. The statistical results of the Two-way ANOVA and one-way ANOVA analysis followed by post Hoc LSD test were obtained using IBM SPSS Version 24 (SPSS, Cary, NC, USA). Values were considered as a criterion for a statistically significant difference; where F value having P< 0.05 was considered significant.

## Results

### Clinical observations, body weight and organ indexes

No mortality was observed in any of the treated groups. Minor loss of furring was the only change detected when rats were treated with each nanoparticle individually. Food and water consumption increased in the ZnO group for 14 days and in other experimental groups from day 14 until the end of the experiment. The MIX group consumed less food compared to other groups.

**Figure 2:**
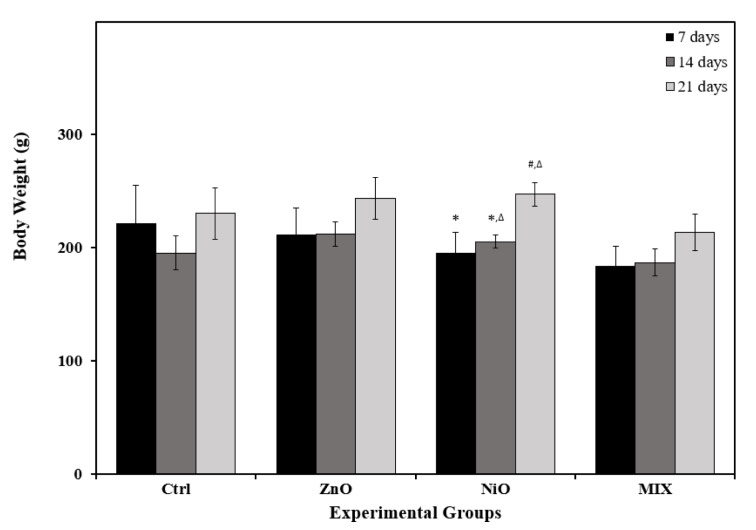
Body weights of the control rats and those administered ZnO-NPs and/or NiO-NPs after 7, 14, and 21 days of administration Data is represented as mean ± standard error. The error bar indicates the standard error of the mean (SEM). Superscript symbols (*, #, ∆) indicate significance within time intervals. The values marked with the same superscript symbol are similar (P>0.05), whereas those marked with different ones are significantly different (P<0.05). Non-significant interaction was observed between time and groups by overall 2-way ANOVA; F=0.308, P>0.05.

Figure 2(Fig. 2) showed a minor reduction in body weight in ZnO and NiO groups on day 7 with insignificant reduction in MIX groups during all time intervals compared to controls. As shown in Table 1(Tab. 1), liver weight decreased significantly (P<0.05) on day 7 in ZnO and NiO groups and throughout the experiment in MIX groups compared with the corresponding control. Furthermore, the liver index decreased significantly (P<0.05) in NiO and MIX groups on day 7 compared to control. On the other hand, kidney weight of NiO and MIX groups were significantly lower (P<0.05) on day 7 compared to Ctrl group. However, a significant gradual increase in kidney weight was observed between time intervals in NiO groups. Kidney indexes were significantly lower in ZnO and NiO groups on day 21 compared to control. No further significant differences in organ weight and indices during other time intervals were observed. 

### Effect of NPs on hematological markers

As shown in Table 2(Tab. 2), in rats receiving *i.p.* administration of ZnO-NPs, RBC indices decreased insignificantly on day 7 compared with the Ctrl group, while a significant increase (P<0.05) in Hb and HCT levels was observed on day 14 as opposed to the Ctrl group. NiO group showed an insignificant decrease in RBC count during 14 days, while Hb and HCT levels did not display any significant change. MIX group revealed an insignificant decrease in RBC, Hb, and HCT levels during 14 days. On day 21, the RBC indices in all experimental groups increased insignificantly compared to the Ctrl group. WBC count in ZnO groups displayed a significant increase (P<0.05) on day 14 while in NiO groups, the values were insignificantly lower than controls and displayed a gradual decrease over time. MIX groups revealed a significant increase in WBC count on day 7 compared to the control whereas the increase on day 14 and 21 was insignificant. Although there were no significant changes in PLT count among all experimental groups, the values increased in ZnO and MIX groups and decreased in NiO groups compared to the control. 

### Effect of NPs on biochemical parameters

#### Effect on liver and kidney functional markers 

**Figure 3:**
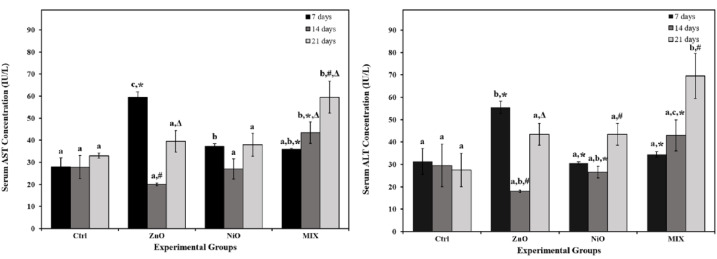
The levels of hepatic functional markers aspartate and alanine aminotransferase (AST and ALT) in serum of the control rats and those administered ZnO-NPs and/or NiO-NPs after 7, 14, and 21 days of administration Data is represented as mean ± standard error. The error bar indicates the standard error of the mean (SEM). Superscript letters (a, b & c) indicate significance within groups. The values marked with the same superscript letter are similar (P>0.05), whereas those marked with different ones are significantly different (P < 0.05). Superscript symbols (*, #, ∆) indicate significance within time intervals. The values marked with the same superscript symbol are similar (P>0.05), whereas those marked with different ones are significantly different (P<0.05). Significant interaction was observed in ALT and AST levels between time and groups by overall 2-way ANOVA; F=8.122, P<0.001 and F=8.698, P<0.001, respectively.

**Figure 4:**
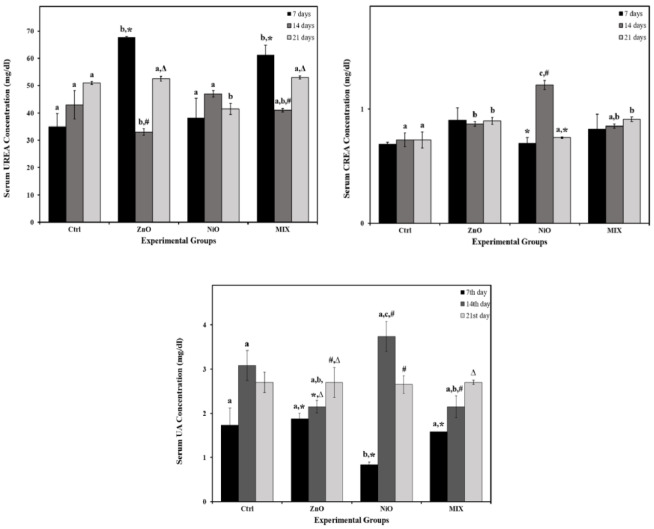
The levels of renal functional markers creatinine (CREA), urea and uric acid (UA) in serum of the control rats and those administered ZnO-NPs and/or NiO-NPs after 7, 14, and 21 days of administration Data is represented as mean ± standard error. The error bar indicates the standard error of the mean (SEM). Superscript letters (a, b & c) indicate significance within groups. The values marked with the same superscript letter are similar (P>0.05), whereas those marked with different ones are significantly different (P < 0.05). Superscript symbols (*, #, ∆) indicate significance within time intervals. The values marked with the same superscript symbol are similar (P>0.05), whereas those marked with different ones are significantly different (P<0.05). Significant interaction (P<0.05) was observed in CREA, UREA and UA levels between time and groups by overall 2-way ANOVA; F=3.884, P=0.008, F=13.721, P<0.001 and F=4.654, P=0.003, respectively.

As shown in Figure 3(Fig. 3), ZnO groups displayed a significant increase (P<0.05) in AST and ALT levels on day 7 compared to controls with no significant alterations in other time intervals. Additionally, NiO groups revealed a significant increase in AST level on day 7 compared to the Ctrl group with no further significant alterations, yet, the highest AST and ALT values were reported on day 21. MIX groups increased gradually and significantly in AST levels on days 14 and 21 and in ALT levels on day 21 compared to all other groups. The interaction of treatment and time was found to be significant (P<0.05) in AST and ALT levels. As shown in Figure 4(Fig. 4), ZnO groups revealed a significant increase in CREA levels on days 14 and 21, and UREA levels during 14 days compared to controls. NiO groups showed a significant increase in CREA on day 14 and a decrease in UREA on day 21 compared to controls. MIX groups showed a significant increase in CREA on day 21 and UREA on days 7 and 21 compared to controls. UA levels were significantly de creased only in NiO group on day 7 compared to control. No further significant alterations were detected in all experimental groups. The interaction of treatment and time was found to be significant (P<0.05) in CREA, UREA and UA levels.

### Effect of NPs on lipid profile

**Figure 5:**
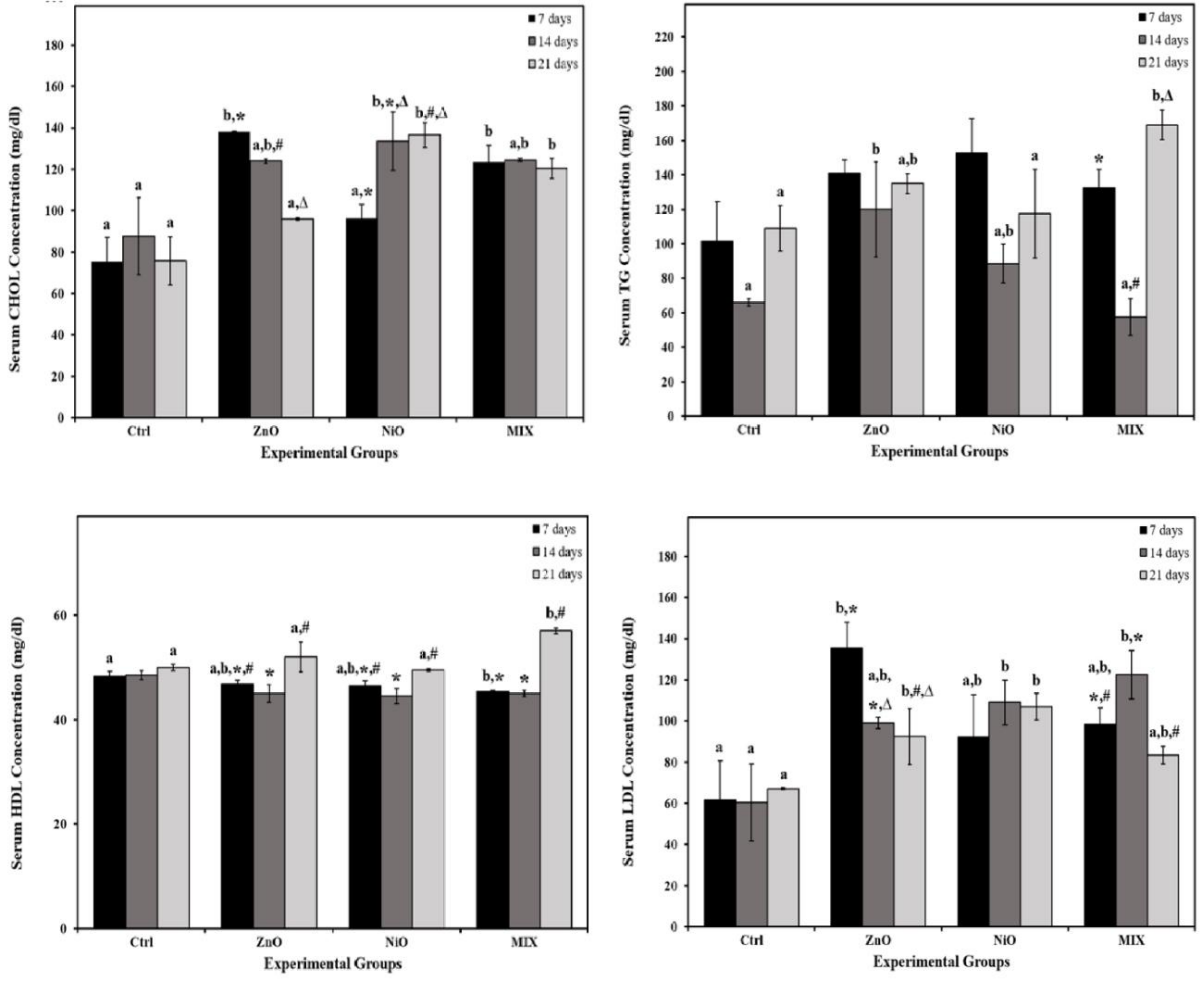
The levels of cholesterol (CHOL), triglyceride (TG) and high and low-density lipoprotein (HDL and LDL) in serum of the control rats and those administered ZnO-NPs and/or NiO-NPs after 7, 14, and 21 days of administration Data is represented as mean ± standard error. The error bar indicates the standard error of the mean (SEM). Superscript letters (a, b & c) indicate significance within groups. The values marked with the same superscript letter are similar (P>0.05), whereas those marked with different ones are significantly different (P < 0.05). Superscript symbols (*, #, ∆) indicate significance within time intervals. The values marked with the same superscript symbol are similar (P>0.05), whereas those marked with different ones are significantly different (P<0.05). Significant interaction was observed in CHOL and HDL between time and groups by overall 2-way ANOVA; F=3.493, P=0.013 and F=4.602, P=0.003 respectively. Non-Significant interaction (P>0.05) was observed in TG and LDL between time and groups by overall 2-way ANOVA; F=2.409 and F=1.928, respectively.

As shown in Figure 5(Fig. 5), compared to control groups, ZnO groups demonstrated a significant increase (p<0.05) in the levels of CHOL on day 7, TG on day 14 and LDL on days 7 and 21, whereas HDL level decreased during 14 days insignificantly. NiO groups displayed a significant increase in CHOL and LDL levels on days 14 and 21 compared to controls. However, TG was insignificantly increased during all time intervals compared to controls. HDL levels decreased insignificantly in NiO groups during 21 days as opposed to the Ctrl groups. On the other hand, MIX groups showed a significant elevation (P<0.05) in CHOL level on days 7 and 21, in TG levels on day 21 and in LDL levels on day 14, whereas HDL levels decreased significantly (P<0.05) on day 7 compared to the control groups. The interaction of treatment and time was found to be significant (P<0.05) in CHOL and HDL levels.

### Effect of NPs on glucose, albumin and total protein

The changes in glucose, albumin and total protein concentrations are displayed in Table 3(Tab. 3). ZnO groups revealed a significant increase (P<0.05) in GLU content on days 14 and 21 compared to Ctrl groups, yet the values revealed to be gradually decreasing with time. GLU concentration displayed a significant increase in NiO groups on day 21 and in MIX groups on days 14 and 21 compared to controls, with levels increasing over time in both groups. Total protein content increased gradually and significantly on day 21 in all experimental groups while ALB content increased significantly on days 14 and 21 in ZnO groups and on day 7 in MIX group compared to controls. No further significant changes were observed in these parameters. The interaction of treatment and time was found to be significant (P<0.05) in ALB level.

### Effect of NPs on antioxidant levels 

**Figure 6:**
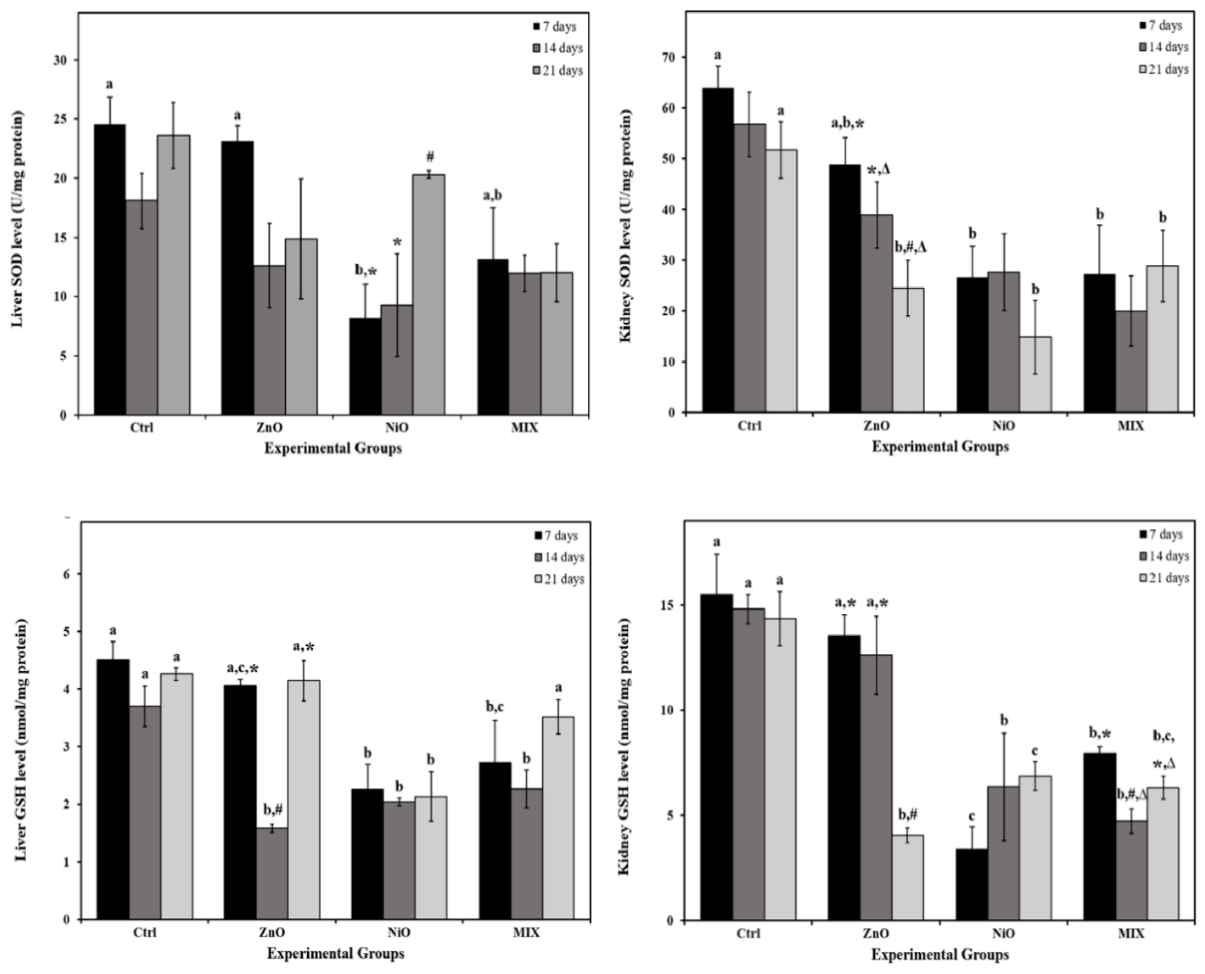
The activity of superoxide dismutase (SOD), and glutathione (GSH) in liver and kidneys of the control rats and those administered ZnO-NPs and/or NiO-NPs after 7, 14, and 21 days of administration Data is represented as mean ± standard error. The error bar indicates the standard error of the mean (SEM). Superscript letters (a, b & c) indicate significance within groups. The values marked with the same superscript letter are similar (P>0.05), whereas those marked with different ones are significantly different (P < 0.05). Superscript symbols (*, #, ∆) indicate significance within time intervals. The values marked with the same superscript symbol are similar (P>0.05), whereas those marked with different ones are significantly different (P<0.05). Non-Significant interaction (P>0.05) was observed in liver and kidney SOD activity between time and groups by overall 2-way ANOVA; F=1.663 and F=0.646, respectively. Significant interaction was observed in liver and kidney GSH content between time and groups by overall 2-way ANOVA; F = 2.893, P=0.029 and F = 6.143, P<0.001 respectively.

As shown in Figure 6(Fig. 6), all groups experienced an insignificant decrease in liver SOD content during the experiment, except for NiO group that displayed a significance on day 7 compared to controls. Liver GSH level also decreased significantly on day 14 in ZnO group, on days 7 and 14 in MIX group and during all time intervals in NiO group compared to controls. In kidneys, SOD activity in ZnO group decreased gradually with time recording a significance only on day 21 compared to the Ctrl group. However, kidney SOD activity decreased significantly on days 7 and 21 in NiO and MIX groups compared to controls. In addition, kidney GSH levels showed a gradual decrease in ZnO groups, which became significant on day 21 compared to the Ctrl group. Furthermore, NiO and MIX groups displayed a significant decrease in kidney GSH content during all time intervals compared to the control, and the values on day 21 where higher than those recorded on day 14. The interaction of treatment and time was found to be significant (P<0.05) in liver and kidney GSH levels.

### Effect of NPs on lipid peroxidation 

**Figure 7:**
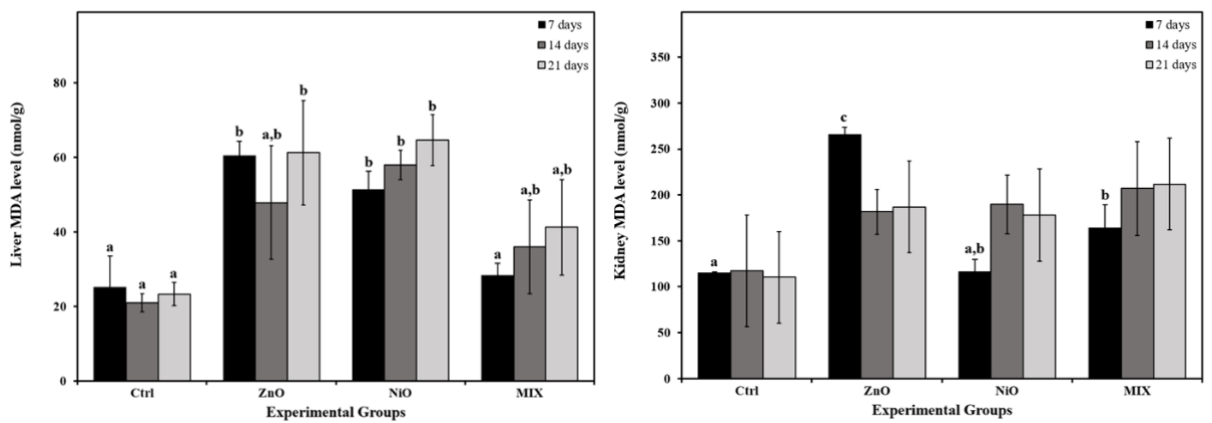
Lipid peroxidation measured by malondialdehyde (MDA) levels in liver and kidneys of the control rats and those administered ZnO-NPs and/or NiO-NPs after 7, 14, and 21 days of administration Data is represented as mean ± standard error. The error bar indicates the standard error of the mean (SEM). Superscript letters (a, b & c) indicate significance within groups. The values marked with the same superscript letter are similar (P>0.05), whereas those marked with different ones are significantly different (P < 0.05). Non-Significant interaction (P>0.05) was observed in liver and kidney MDA levels between time and groups by overall 2-way ANOVA; F=0.372 and F = 1.163, respectively.

MDA concentration in the liver increased significantly in ZnO groups on days 7 and 21, and in NiO group in all time intervals compared to controls. Further, the values in NiO groups were shown to be increasing with time. Also, MIX groups displayed an insignificant gradual increase in liver MDA levels during the experiment compared to controls. On the other hand, kidney MDA level increased significantly on day 7 in ZnO and MIX groups compared to the Ctrl group. No significant differences were observed in other time intervals. The results of liver and kidney MDA levels are illustrated in Figure 7(Fig. 7).

### Histopathological findings

**Figure 8:**
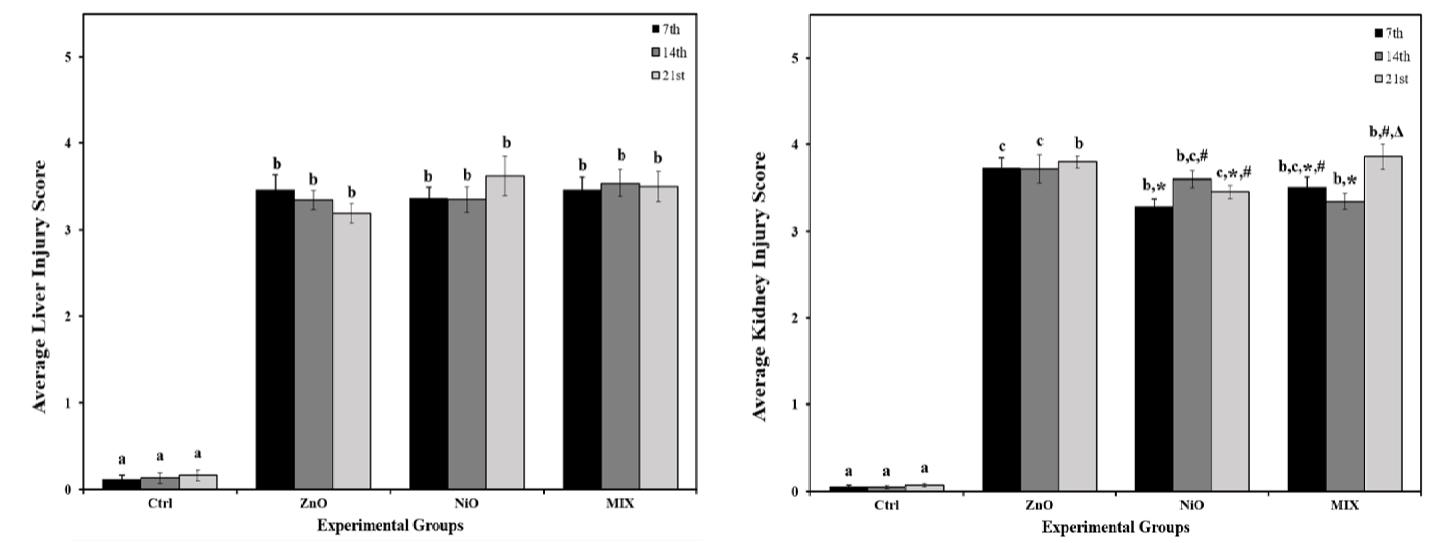
Average liver and kidney injury score in control rats and those administered ZnO-NPs and/or NiO-NPs after 7, 14, and 21 days of administration Data is represented as mean ± standard error. The error bar indicates the standard error of the mean (SEM). Superscript letters (a, b & c) indicate significance within groups. The values marked with the same superscript letter are similar (P>0.05), whereas those marked with different ones are significantly different (P < 0.05). Superscript symbols (*, #, ∆) indicate significance within time intervals. The values marked with the same superscript symbol are similar (P>0.05), whereas those marked with different ones are significantly different (P<0.05). Non-Significant interaction (P>0.05) was observed in average liver injury score between time and groups by overall 2-way ANOVA; F=0.688. A significant interaction was observed in average kidney injury score between time and groups by overall 2-way ANOVA; F=2.399 and P=0.032.

The effects of ZnO-NPs and NiO-NPs toxicities were examined at different time intervals (7, 14, and 21 days). Most organs, except for the liver, did not exhibit apparent gross pathological alterations when compared to controls. ZnO-NPs treated liver revealed severe to moderate white granulomatous patches on their surface after 7 and 14 days respectively. As compared to ZnO-NPs, the liver in NiO-NPs on day 21 was severely inflamed with the presence of marked granulomatous inflammation among the liver lobes. Moreover, MIX treated tissues did not show any morphological abnormalities. The H&E stained histological slides of liver and kidneys were semi-quantitatively assessed for histological alterations and compared with the controls. The scoring of histopathological damage and alteration is shown in Figure 8(Fig. 8), revealing the extent of average liver and kidney injury among treated groups at different time intervals, while showing significant interaction (P<0.05) between time and treatment in kidney injury score.

**Figure 9:**
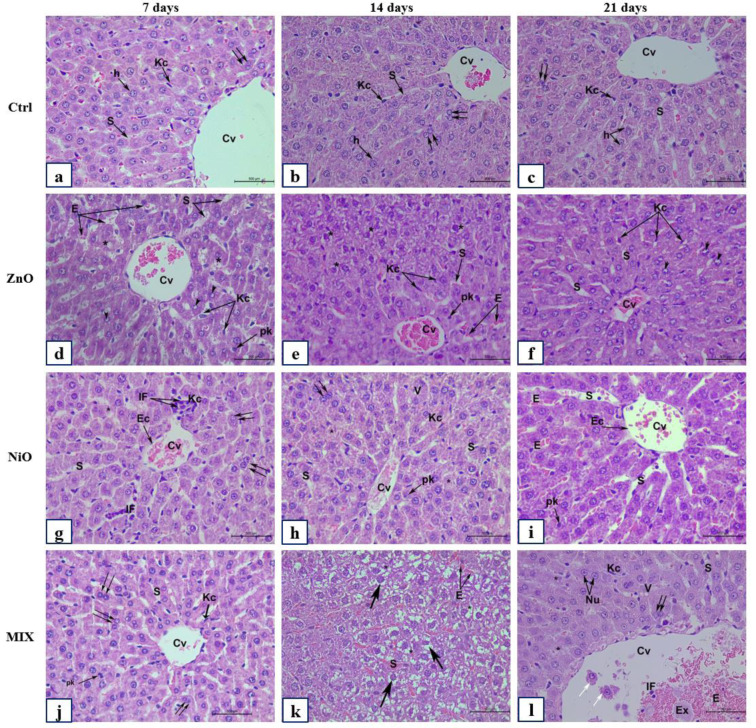
The histological changes of the liver (pericentral area) of control and experimental groups after 7, 14, and 21 days. Representative photomicrographs of serial sections of liver tissue from rats in Ctrl (a-c), ZnO (d-f), NiO (g-i), and MIX (j-l). (a-c) Ctrl group illustrating classical hepatic trabecular architecture with polygonal shape hepatocytes (h), binucleated cells (double arrow), central vein (Cv), sinusoids (S) with Kupffer cells (Kc). (d) ZnO group after 7 days showing congested (Cv), dilated (S) with numerous (Kc) and endothelial cells (Ec), hepatocytes with vesicular nuclei (arrowhead). (e-f) ZnO groups showing degraded hepatocytes with pyknotic nuclei (pk). (g-i) NiO group demonstrating mononuclear cellular infiltration (IF), increased (Kc), (pk), ballooning of hepatocytes with cytoplasmic vacuolization (V). (j) MIX group after 7 days showing normal liver architecture; (k-l) MIX group illustrating hepatocyte cytoplasmic micro steatosis (arrow), centralization of nucleoli (Nu), congested (S) with erythrocytes (E), congested (Cv) with cellular shedding in the lumen (white arrow), and IF. Total magnification 400X, scale bar: 500 µm, H&E stained sections

**Figure 10:**
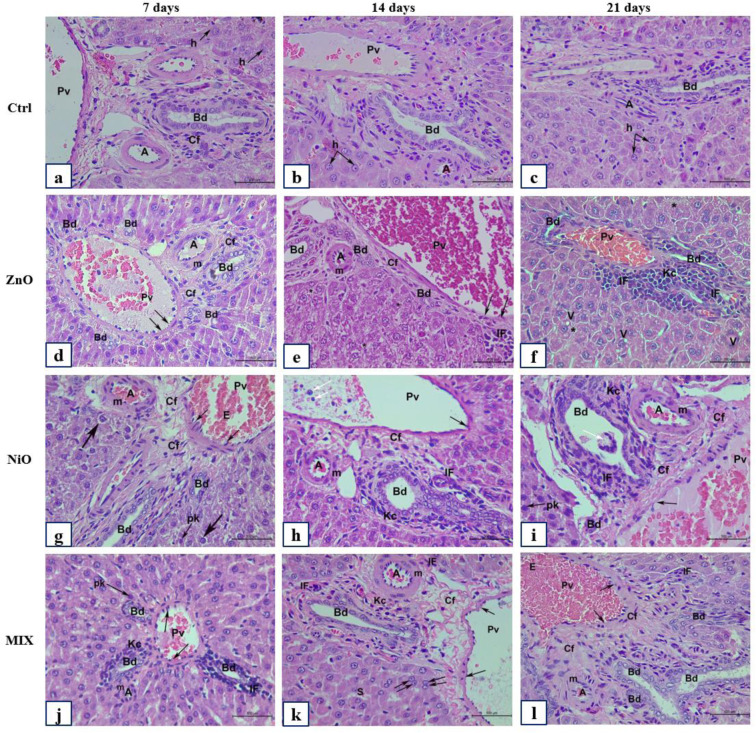
The histological changes of the liver (periportal area) of control and experimental groups after 7, 14, and 21 days. Representative photomicrographs of serial sections of liver tissue from rats in Ctrl (a-c), ZnO (d-f), NiO (g-i), and MIX (j-l). (a-c) Ctrl group showing portal vein (Pv), bile duct (Bd), hepatic artery (A), regular periportal hepatocytes (h). (d) ZnO group after 7 days showing congested (Pv), extensive elongation of endothelial lining (arrow), moderate fibrosis with connective tissue fiber (Cf), congested (A) with thickened muscular layer (m), proliferation of (Bd). (e-f) ZnO groups with congested (Pv), periportal inflammatory cellular infiltration (IF), numerous (Kc), hydropic degeneration of hepatocytes showing ballooning (*) with cytoplasmic vacuoles (V). (g) NiO group after 7 days demonstrating hepatocytes with pk and signs of cytoplasmic microsteatosis (arrow). (h-i) NiO group demonstrating severely congested (Pv) with cellular shedding in the lumen (white arrow), elongation of endothelial lining (arrow), (Bd) proliferation surrounded with periportal (IF), extensive masses of (Kc), (Cf), reduced sized hepatocytes with (pk). (j) MIX group after 7 days showing moderate pathological features; (k-l) MIX group illustrating moderate to severe histological alteration. Total magnification 400X, scale bar: 500 µm, H&E stained sections

**Figure 11:**
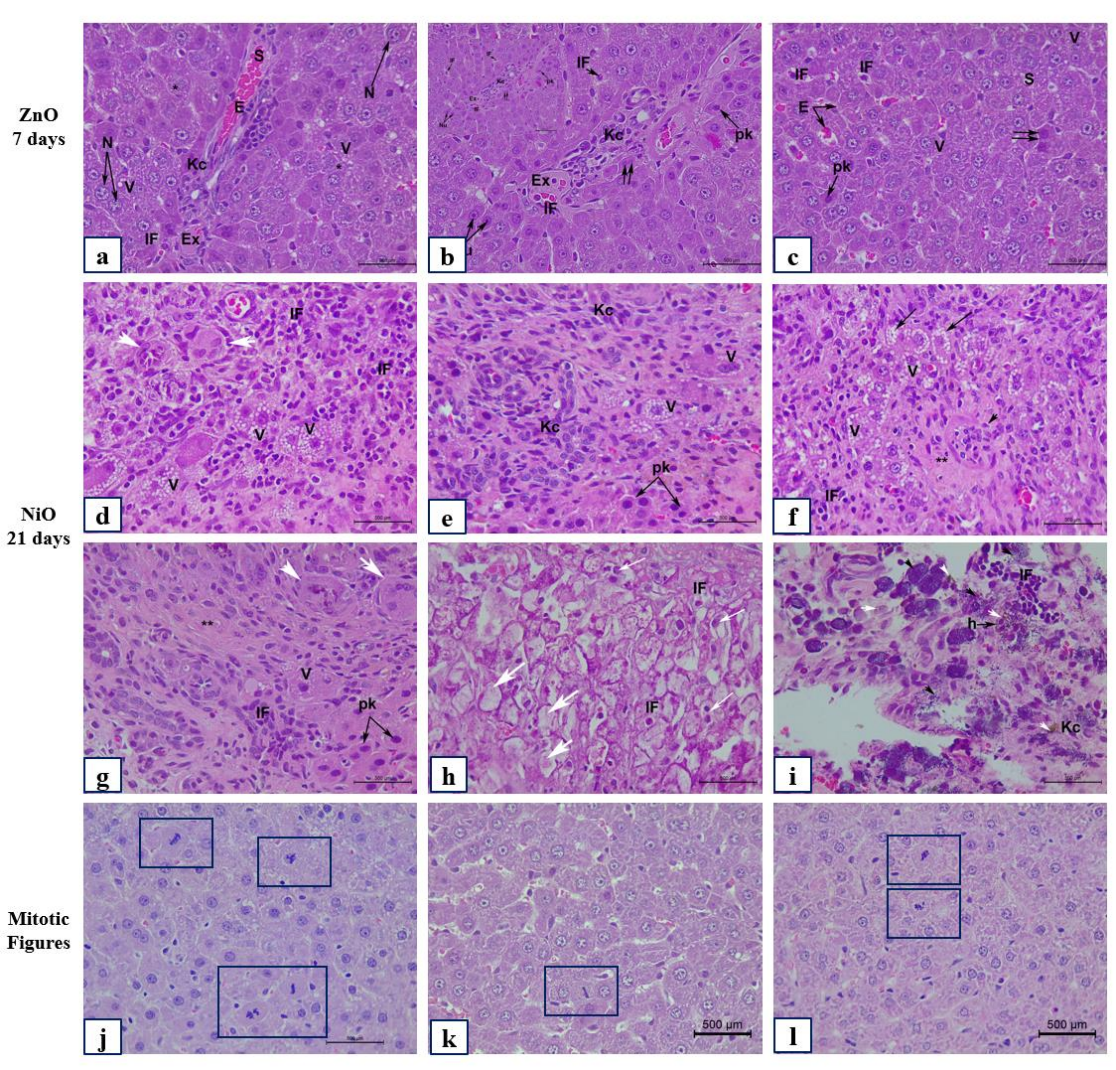
Additional histological and pathological changes of the liver of ZnO and NiO experimental groups. Representative photomicrographs of serial sections of liver tissue from rats in ZnO (a-c) after 7 days, NiO (d-i) after 21 days. (j-l) demonstrating mitotic figures control and experimental group. (a-c) ZnO group showing congested and dilated sinusoids (S), hepatocytes with large nuclei (N) and ballooned cytoplasm with vacuoles (V), numerous (Kc) hyperplasia, IF, and signs of pyknosis (pk) in binucleated cells (double arrow). (d-g) NiO group showing ballooning of hepatocytes with foamy vacuolated cytoplasm (V), severe inflammatory cellular infiltration (IF) forming granulomatous inflammation (Giant cell reaction) (white arrow head), fibrosis (**), necrotic foci with signs of pyknosis (pK), numerous (Kc) showing hyperplasia (arrowhead), hepatocytues ballooning (*) with cytoplasmic vacuoles (V). (h) NiO group demonstrating focal fatty changes with macrosteatosis (thick white arrow) and microsteatosis (Thin white arrow). i) NiO group displaying mineralization at the level of the portal space with intra- and intercellular basophilic deposits (arrowhead) are greatly associated with numerous (IF). Observe, yellow to brown granulated pigmentation (white arrowhead) deposited among hepatocytes and Kupffer cells (Kc). (j-l) mitotic figures in Ctrl group (j); ZnO group (k) after 21 days; MIX group (l) after 7 days. Total magnification 400X, scale bar: 500 µm, H&E stained sections

Examination of H&E stained liver sections of the control group (Ctrl) after 7, 14, and 21 days are represented in Figures 9(a-c)(Fig. 9), 10(a-c)(Fig. 10) and 11(a-c)(Fig. 11). Liver and renal tissue in the study groups of male Sprague Dawley rats for both ZnO-NPs and NiO-NPs *i.p.* administration showed moderate to severe histopathological changes in a time-dependent manner. At 200 mg/kg concentration of ZnO-NPs, histological observation and analysis under light microscopy showed significant morphological changes in the liver architecture as well as severe damage to the hepatic tissue on day 7 when compared to the untreated control group. Figure 9d(Fig. 9) showed changes in hepatic lobule architecture with a marked loss of trabecular architecture of the hepatocytes surrounding a highly congested and dilated central vein. A well-marked hydropic degeneration of hepatocytes was highly manifested; where most hepatocytes appeared hypertrophied and exhibited ballooning and vacuolization of the cytoplasm (Figures 9d(Fig. 9), 11a(Fig. 11)). Densely stained pyknotic nuclei were frequently detected at certain foci of hepatic necrosis (Figures 9d(Fig. 9), 11a(Fig. 11)). Besides, a moderate mononuclear cellular infiltrate was seen in the portal area (Figure 11a(Fig. 11)). The portal area exhibited a highly widened and congested portal vein, proliferation of bile duct, and necrotic foci among hepatocytes with inflammatory cellular infiltration (Figure 10d(Fig. 10)). Besides, the portal vein appeared dilated and congested with a great elongation of its endothelial lining (Figure 10d(Fig. 10)) accompanied with moderate fibrosis (Figure 10d(Fig. 10)). In addition, the hepatic artery was severely congested and showed thickening of its muscular layer (Figure 10d(Fig. 10)). Kupffer cell activation was highly observed after 7 days, where Kupffer cells were severely and numerously clustered and aggregated to form hyperplasia (Figure 11a-d(Fig. 11)) within the dilated and congested sinusoids. Examination of the liver sections of ZnO groups after 14 days showed nearly the same histological features as those present after 7 days but with moderate grading pathological features (Figures 9e(Fig. 9), 10e(Fig. 10)). However, after 21 days of observation, ZnO group showed a slight improvement compared to 7 and 14 days (Figure 9f(Fig. 9)); where the liver architecture was preserved minimally and slightly restored to normal. Though many binucleated hepatocytes were significantly detected along with mitotic figures (Figure 11k(Fig. 11)), yet, a large number of hepatocytes showed vacuolated cytoplasm and vesicular nuclei, as well as, mildly dilated sinusoids (Figure 9f(Fig. 9)). Besides, portal area (Figure 10f(Fig. 10)) showed less dilated and mildly congested portal vein with a marked reduction in the inflammatory cellular infiltrate in comparison to ZnO groups after 7 and 14 days of observation (Figure 11c(Fig. 11)).

On the other hand, NiO-NPs administration displayed severe morphological changes in the liver tissues, similar to those described in ZnO group. These changes were graded as moderate to severe after 7 and 14 days respectively (Figures 9g-h(Fig. 9) and 10g-h(Fig. 10)). Surprisingly, after 21 days of NiO-NPs administration severe hepatic histopathological changes as well as marked cellular degenerative changes were shown in the liver trabecular architecture when compared to their corresponding groups after 7 and 14 days, and similarly with their corresponding observed in ZnO and Mix group (Figures 9i(Fig. 9), 10i(Fig. 10)). These changes include severe congestion and dilation of portal vein. Besides, most of the bile ducts were highly congested and revealed signs of cellular shedding and blood leakage into the lumen. Moreover, necrotic foci in between hepatocytes were observed accompanied by marked masses of inflammatory cell infiltration and exudation invasion among portal vessels (peri-portal inflammatory cellular infiltration). Hepatocytes exhibited severe fatty changes including signs of micro- (Figure 11d-h(Fig. 11)) and macrosteatosis (Figure 11h(Fig. 11)). Some hepatocytes revealed signs of “Pigmentation”, where pale yellow to deep granular brown pigments was seen deposited in some hepatocytes as well as in Kupffer cells (Figure 11i(Fig. 11)). Moreover, a large number of hepatocytes showed “Mineralization”, an additional pathological feature manifested by an enormous deposition of intra- and extracellular basophilic deposits (Figure 11i(Fig. 11)). Strikingly, the most severe pathological change was the marked increase in mononuclear cell infiltration forming “granulomatous inflammation”. In addition, numerous bi- and multinucleated hepatocytes were detected exhibiting an intense “Giant cell reaction” characterized by liver parenchymal inflammation (Figure 11d-g(Fig. 11)). Furthermore, liver tissues received mixed administration, though showed preservation of the liver architecture after 7 days with significantly observed mitotic figures (Figure 11l(Fig. 11)), yet moderate to severe morphological changes that were significantly manifested after 14 and 21 days as compared to the control group and ZnO group (Figures 9j-l(Fig. 9), 10j-l(Fig. 10)) and illustrated in Figure 8(Fig. 8). 

**Figure 12:**
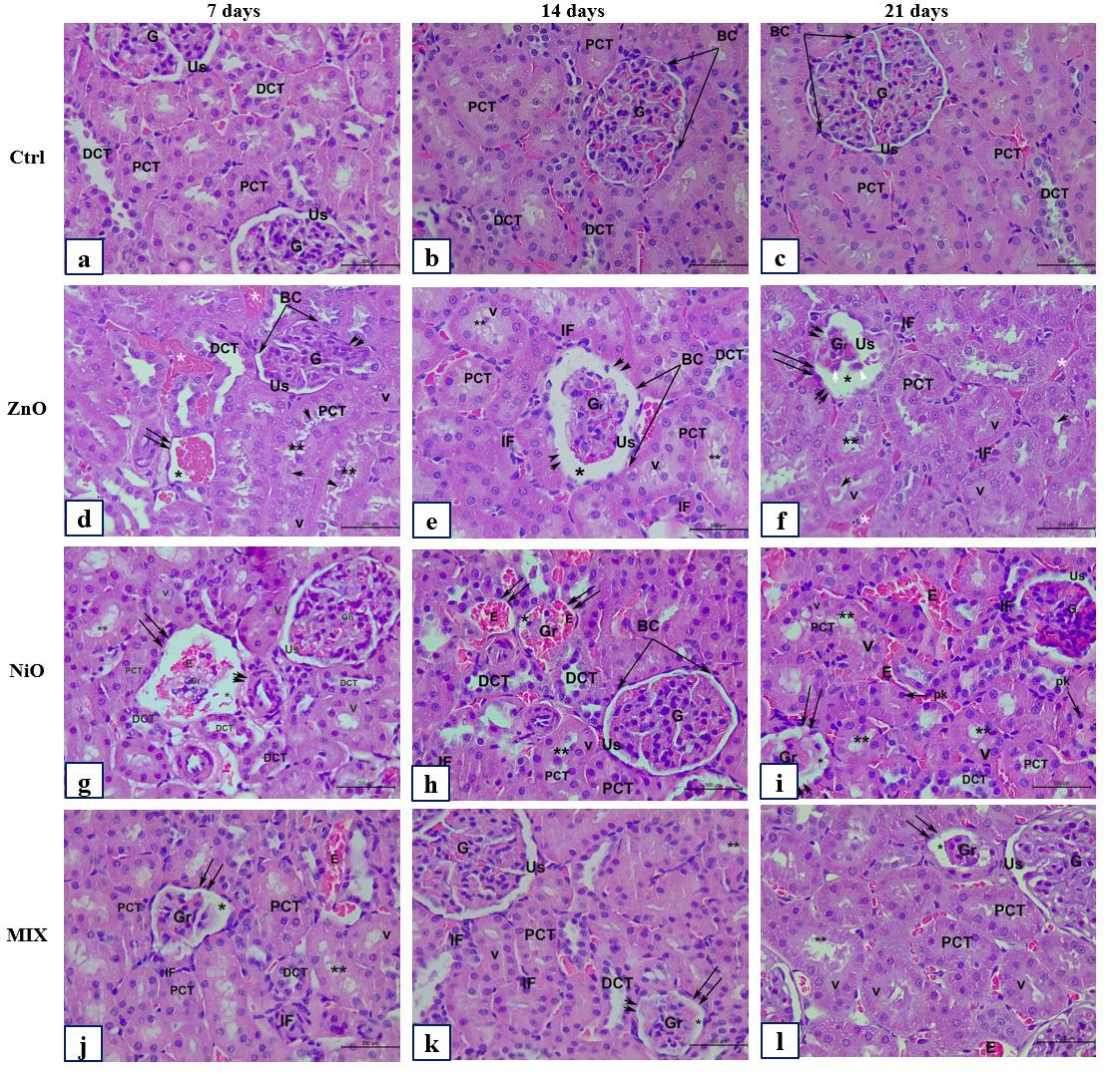
The histological changes of the kidney of control and experimental groups after 7, 14, and 21 days. Representative photomicrographs of serial sections of kidney tissue from rats in Ctrl (a-c), ZnO (d-f), NiO (g-i), and MIX (j-l). (a-c) Ctrl group showing glomerulus (G), regular urinary space (Us), squamous cells lining Bowman's capsule (BC), proximal convoluted tubule (PCT) with brush border, and distal convoluted tubule (DCT). (d-f) ZnO group showing mild, moderate to severe pathological features respectively. (f) Illustrating atrophied renal corpuscle (double arrow), with reduced glomerulus (Gr), dilatation of the US (*), desquamated BC (double arrowhead), peritubular IF, damaged PCT with cellular shedding (arrowhead) and loss of brush borders (**), cellular vacuolization (V), irregular DCT. (g-i) NiO group demonstrating pathological alteration from severe to mild respectively. (g) Demonstrating degenerating and congested renal corpuscle with complete loss of G, and squamous lining of BC (double arrowhead). (h-i) note reduced glomerulus (Gr), with congested erythrocytes (E), PCT with loss of brush borders (**), and cytoplasmic vacuolization (V). (j-l) MIX group showing moderate pathological features; (k-l) MIX group illustrating severe to moderate histological alteration. Reduced renal corpuscle (double arrow), (Gr), Dilated urinary space (*), peritubular infiltration (IF). Total magnification 400X, scale bar: 500 µm, H&E stained sections

Kidney tissues of the control groups are represented in Figure 12 (a-c)(Fig. 12). The intraperitoneal injection of 200 mg/kg of ZnO-NPs revealed several renal pathological changes when compared to the untreated Ctrl group. These changes were graded as mild, moderate, and severe after 7, 14, and 21 days respectively. In Figure 12d(Fig. 12), after 7 days of ZnO-NPs administration, the pathological feature detected include congestion of the glomeruli with marked glomerular atrophy (Loss of glomerular cell) at certain foci. Besides, the Bowman's capsule is damaged, and desquamated; whereas the proximal convoluted tubules (PCT) exhibited signs of necrosis where cells were hypertrophied and vacuolated. Additionally, moderate and severe pathologic changes were detected after 14 and 21 days of ZnO-NPs administration including marked shrinkage of glomeruli (Figure 12e-f(Fig. 12)) with a severe widening and dilatation of the urinary space and desquamated epithelium, degeneration and loss of some glomeruli, distortion of renal tubular architecture, loss of PCT brush border, increased inflammatory cell infiltration and congestion among renal corpuscles and tubules.

However, NiO-NPs administration displayed similar renal histopathological features to those present in ZnO group. The changes were graded as moderate and moderately- severe after 7 and 14 days respectively (Figure 12g-h(Fig. 12)); and included: necrosis of glomeruli with severe signs of congestion, atrophy, and loss; degenerated and desquamated necrotic renal tubules with cytoplasmic vacuolization, ballooned cells with pyknotic nuclei, loss of brush borders; as well as inflammatory cells infiltrating in the interstitium. Yet, the histopathological changes in the kidneys after 14 days of injection were more severe than at day 7 (Figure 12h(Fig. 12)). Conversely, NiO-NPs administration after 21 days revealed mild changes in the renal cortical architecture and structures compared to those observed in Ctrl group (Figure 12i(Fig. 12)). Nevertheless, mild to moderate pathological changes including; glomerular congestion, irregularities of renal tubules morphology with vacuolization, congestion, and inflammatory cell infiltration were still manifested in such group.

H&E stained kidney sections of Mixed administration exhibited pathological features that were significantly moderate and severe when compared to ZnO group; as well as to the Ctrl & NiO groups; respectively after 7 days. The most severe morphological changes after 7 days were the degenerated and congested glomeruli, damaged vacuolated renal tubules, and inflammatory cell infiltration (Figure 12j(Fig. 12)). The renal histological features of the MIX group were mild and moderate after 14 and 21 days, where kidney sections displayed normal renal cortical and structural arrangement, yet it retained minimal pathological features (Figure 12k-l(Fig. 12)). 

See also the Supplementary data.

## Discussion

ZnO-NPs and NiO-NPs are widely used in various commercial and industrial products, and their continuous exposure raises concerns about their potential adverse effects on human health (Sousa et al., 2018[61]; Kong et al., 2019[39]). Hence, it is crucial to expand our understanding concerning the adverse effects that they could exert in biological systems. In this study, we aimed to investigate the *in vivo* acute toxicity of ZnO-NPs (≃ 68 nm) and/or NiO-NPs (≃ 29 nm) following their administration in SD rats, through *i.p.* route, which is efficient and rapid, and since most nanotoxicity studies have focused on alternate routes of exposure. ZnO-NPs and NiO-NPs were previously synthesized using the co-precipitation method, and their various physiological characteristics were taken into consideration, as they could have a major influence on how the NPs behave and end up inside biological systems (Zein et al., 2020[73]). This study primarily focused on the liver and kidneys since these organs are targets for NP toxicity (Ajdary et al., 2018[7]).

Body and organ weight measurements are widely used to assess the potential adverse effects of drugs on animals (Lazic et al., 2020[40]). The present study demonstrated that ZnO-NPs and NiO-NPs administration separately, induced a momentous decline in body weight, organ weights, and their indices. Additionally, the reduction in organ weights was more prominent in liver and kidneys of NiO groups, indicating that NiO-NPs might be more potent stressors on organs. Notably, NPs co-exposure decreased organ weights and body weight gain across all time periods. Shaban et al. (2022[58]) claim that the harmful effects of NPs on organs induce a reduction in their weight, which subsequently reduce body weight. In addition, the anabolic metabolism of the treated animals, the anti-digestion effect, or the decreased appetite brought on by the administration of NPs could be attributed to the reduction in body weight (Ramadan et al., 2022[54]). Our results are consistent with Ashajyothi et al. (2018[16]) who reported a momentous reduction in body weight after recei-ving ZnO-NPs. Similarly, Yousef et al. (2019[72]) showed a reduced body weight gain following the co-exposure to ZnO-NPs and Al_2_O_3_-NPs, compared to their individual exposure. Moreover, Ali (2019[10]) also showed the same alterations when NiO-NPs were administered with Co_3_O_4_-NPs.

The hematologic system possesses a high predictive value for body toxicity (van der Zande et al., 2012[68]). RBC, Hb and HCT levels decreased initially, before remarkably increasing at the end of the experiment. The drop might be an indication of RBC destruction or NPs interference with erythropoiesis, and the subsequent increase in RBC and Hb can be seen as a feedback mechanism, and the greater HCT level suggests an increase in blood viscosity (Shah, 2006[59]; Morsy et al., 2016[47]). On the other hand, WBC and PLT counts increased in ZnO and MIX groups and decreased in NiO groups. While elevated WBC counts indicate that NPs induced an immunological response, elevated PLT counts can be attributed to either increased platelet synthesis or a decrease in their removal by the damaged spleen (McMillan, 2009[44]). However, blood leakage from vessel walls or ROS-induced cellular damage could be the reason for their decline (He et al., 2020[31]). Our findings are in line with those of Sudhakaran et al. (2020[63]) who revealed that treatment of ZnO-NPs intraperitoneally induced the same changes in hematological indices. In two separate experiments, administration of mixtures of ZnO-NPs and CuO-NPs and NiO-NPs and Mn3O4-NPs both resulted in the same outcomes as MIX groups (Katsenlson et al., 2015[37]; Attia et al., 2019[17]). 

The functional status of important organs, including the liver and kidneys is provided by assessing biochemical parameters (Kong et al., 2019[39]). The findings of the present study revealed an increase in AST, ALT CREA and urea levels in a time-dependent manner. Whether provided alone or in combination, NiO-NPs induced a stronger and more permanent effect on liver enzymes while gradually diminishing its effect in kidney. On the other hand, the individual exposure to ZnO-NPs exhibited an ongoing effect on the kidneys while gradually lowering their toxicity in the liver. Elevated levels of liver enzymes might be a result of enzymatic leakage and increased permeability during liver cell damage (Sudhakaran et al., 2020[63]). Additionally, a rise in urea and CREA concentrations indicates that glomerular filtration is impaired (Said et al., 2022[56]). Tissue observations of liver and kidney in the present study further support the abovementioned hypothesis. Interestingly, a reduction in urea concentrations was detected at the end of the experiment in NiO groups which might be attributable to impaired liver function (Glavind et al., 2016[30]). Moreover, UA levels were found to decrease in the present study, which might be explained on the basis of oxidative stress generation upon NPs administration since UA is associated with the activation of the xanthine oxidoreductase inhibitor that lowers its level exerting a type of protection in situations associated with oxidative stress (Glantzounis et al., 2005[29]). The present findings are consistent with several studies in which administration of ZnO-NPs and NiO-NPs, either individually or in combination with other NPs, provoked the same changes in liver and kidney functional indicators (Katsenlson et al., 2015[37]; Yousef et al., 2019[72]; Sudhakaran et al., 2020[63]). 

Zhang et al. (2021[74]) stated that lipid metabolism is directly associated with liver injury. Our results revealed that CHOL, TG and LDL levels increased and HDL level decreased, a condition known as “Dyslipidemia” (Lynda et al., 2011[41]). It is suggested that lipid peroxidation, which is correlated with both CHOL and TG, can be induced by NPs as a result of excessive ROS production and membrane destruction which eventually increases lipid contents in blood (Ademowo et al., 2017[4]). These changes were primarily noticeable on day 7 in ZnO groups and days 14 and 21 in NiO, and MIX groups. Further, a momentous decline in TG content was observed on day 14 in NiO group and might be attributed to the immense use of these molecules as an energy supply for cells to withstand toxicity (Ali, 2019[10]). Our findings matched those of Moatamed et al., (2019[45]) who demonstrated that *i.p.* injection of ZnO-NPs resulted in similar changes in lipid profile of treated rats. Similarly, NiO-NPs, as reported by Zhang et al. (2021[74]) also caused dysregulation in lipid and fatty acids, suggesting liver injuries.

Glucose is considered the primary source of energy for the activities of cells under stress (Bélanger et al., 2011[18]). The increased glucose level in the present study might be attributed to ZnO-NPs and NiO-NPs ability to induce an imbalance in glucagon and cortisol hormones with a decrease in insulin levels resulting in a hyperglycemic response (Cartañà and Arola, 1992[21]; Alkaladi et al., 2020[13]). Additionally, NPs can alter insulin receptors, which inhibits the ability of the insulin substrate to combine with molecules, resulting in a subsequent glucose accumulation in the blood (Hu et al., 2015[34]). Ali (2019[10]) confirmed our results by reporting that the mixture of NiO-NPs and Co_3_O_4_-NPs enhanced the glucose level. Our results contradicted those reported by Slama (2015[60]), who showed a decrease in glucose levels following ZnO-NPs administration.

ALB content increased following exposure to ZnO-NPs alone or in combination with NiO-NPs, whereas NiO-NPs, given separately, decreased ALB levels. On the other hand, total protein content was insignificantly altered initially before remarkably increasing on day 21 in all experimental groups. The elevated ALB levels as observed in ZnO groups could be a sign of kidney damage (Tizhe et al., 2014[67]). Additionally, NiO-NPs induced hepatocellular damage that results in inhibition of bioprotein synthesis may be responsible for the drop in ALB levels seen in NiO groups, which was evident by the increased activities of liver enzymes (Bernardi et al., 2012[19]). Our results were consistent with those reported by Moatamed et al. (2019[45]) showing an increase in ALB concentration with an insignificant reduction in TP level following administration of ZnO-NPs. Ali (2019[10]) also confirmed our findings by reporting almost the same alterations in ALB and TP levels following NiO-NPs and/or Co_3_O_4_-NPs administration. However, Yousef et al. (2019[72]) contradicted our findings by reporting decreased ALB and TP levels following the oral co-exposure of ZnO-NPs and Al_2_O_3_-NPs in rats.

ROS generation and subsequent oxidative stress are well-known mechanisms of NPs toxicity. The overproduction of free radicals or the depletion of antioxidant components such as SOD and GSH indicates the failure to retain the cellular redox equilibrium which subsequently increases MDA levels that interacts with macromolecules generating oxidative stress (Phaniendra et al., 2015[52]). The present study indicated a decrease in antioxidant's levels SOD and GSH in liver and kidney tissues of rats treated with *i.p.* injections of ZnO-NPs and/or NiO-NPs, as well as an increase in MDA level. When compared to ZnO-NPs, NiO-NPs showed higher oxidative damage, which gradually increased with time in the liver while decreasing in the kidney. Similarly, Yousef et al. (2019[72]) demonstrated that SOD and GSH levels reduced and MDA increased in liver and kidneys, following the administration of ZnO-NPs and/or Al_2_O_3_-NPs in rats. Further Ali and Mohamed (2019[12]) also confirmed our results by reporting a remarkable reduction in hepatic and renal SOD and GSH with increased MDA levels in rats given NiO-NPs and/or Co_3_O_4_-NPs. The present findings are supported by the pathological lesions observed in histological analysis as well as the biochemical alterations in the present work.

Histological observations of tissue sections in all experimental groups confirmed the time-dependent alterations observed in all variables. The findings revealed typical histological alterations in liver, which includes, hydropic degeneration and cytoplasmic vacuolization, sinusoidal dilatation and congestion, Kupffer cells hyperplasia and pyknosis with focal necrotic areas. The kidneys depicted glomerulus atrophy and loss, dilated urinary space, dense mesangial cells, tubular necrosis, brush border loss as well as intratubular inflammation. Over time, liver alterations in ZnO group were less severe, displaying signs of hepatoprotective activity such as binucleation and mitotic figures, while renal histological alterations became more apparent, suggesting that ZnO-NPs are mainly accumulated in renal tissues. In contrast, the liver tissues of the NiO groups were more severely affected, displaying additional pathological lesions including fatty changes, granulomatous deposits, bile duct proliferation, mineralization, and pigmentation, indicating that the liver had been subjected to chronic inflammation, hepatic necrosis, and oxidative damage by lipid peroxidation (Abdelhalim and Jarrar, 2012[3]). MIX groups also demonstrated progressive liver damage, although to a lesser extent than NiO groups, suggesting a potential late effect of co-exposed NPs. In kidneys, the severity decreased with time in NiO and MIX groups. This further supports the hypothesis that the NPs in NiO and MIX groups might be transported to the liver for elimination to reduce the amount filtered in the kidneys. These results are in agreement with several previous studies that reported the same histological alterations in liver and kidneys following ZnO-NPs and NiO-NPs administration (Katsnelson et al., 2015[37]; Ashajyothi et al., 2018[16]; Dumala et al., 2019[26]; Al-Al Zerjawe and Al-Bairuty, 2020[9]; Ali et al., 2021[12]). 

To our knowledge, this is the first report of *in vivo* toxicity effect of co-exposure to ZnO-NPs and NiO-NPs intraperitoneally in rats. The combined effect of NPs can be explained based on the alternating patterns of their absorption, distribution, accumulation, and elimination, and the metal ions released after their ionization may be responsible for the different patterns of toxicity that they induce (Ganguly et al., 2018[27]). At the beginning of the experiment, the co-exposed NPs reduced the adverse outcomes of their individual exposure in nearly all parameters. Several investigations demonstrated that Zn^2+^ induce an antagonistic effect against Ni^2+^, which might be brought on by competition between these ions for binding sites on proteins and carriers or Zn' stimulation of metallothionine synthesis that promotes Ni uptake (Šulinskienė et al., 2015[64]; Onodera et al., 2018[50]). However, the co-exposure effect of heavy metals is unpredictable and even contradictory. In the present work, ZnO-NPs' antagonistic effect gradually diminished with time, and a synergistic effect appeared at the end of the study. Herkovits et al. (2000[33]) demonstrated that medium concentrations of Zn enhance Ni toxicity and produce synergism since these concentrations of Zn increase the uptake of Ni. However, with higher concentrations, a protective effect of Zn appears against Ni, which can be related to their competition for the uptake. This highlights the idea that the concentration of Zn in the mixture appears to be the key determinant of the relationship between doses and responses caused by Ni-Zn interactions and should be taken into consideration in future studies.

## Conclusion

To conclude, our results showed that exposure of rats to ZnO-NPs and/or NiO-NPs via intraperitoneal injection altered biological indices, impaired antioxidant status, and induced tissue damage. NiO-NPs were revealed to be more potent toxicants than ZnO-NPs. Furthermore, NiO-NPs was shown to induce a higher level of toxicity in the liver than ZnO-NPs, which was mostly damaging to the kidneys. Additionally, the combined exposure of ZnO-NPs and NiO-NPs initially exhibited less remarkable disturbances, indicating a relative antagonistic action, while inducing a synergistic effect in most variables, at the end of the study, compared to NPs individual effect. Therefore, it is recommended to apply mixtures of ZnO-NPs and NiO-NPs in their widely distributed applications, rather than their individual NPs, during short time intervals, using adequate concentrations of ZnO-NPs, with a particular attention to NPs' size, since the effect of both NPs depends on it. Additional research involving different concentrations of both NPs are required in order to understand the mechanisms involved in the co-exposure of these NPs and to establish a conclusion regarding their synergistic/antagonistic effect. Such studies would provide valuable insights for the development of guidelines and regulations to ensure the safe use of these nanoparticles, individually or in mixtures, across various applications.

## Notes

Noura S. Abouzeinab and Mahmoud I. Khalil (Department of Biological Sciences, Faculty of Science, Beirut Arab University, Beirut, Lebanon and Molecular Biology Unit, Department of Zoology, Faculty of Science, Alexandria University, Alexandria, Egypt; Phone: +96181912014, E-mail: m.khalil@bau.edu.lb) contributed equally as corresponding author.

## Declaration

### Acknowledgments

The authors wish to acknowledge Jamalat Boukhari for providing the ZnO and NiO nanoparticles, Department of Physics, Faculty of Science, Beirut Arab University, Beirut, Lebanon.

### Author contributions

Noura S. Abouzeinab planned and developed methodologies, performed the animal dosing and handling, histological procedures, description, and morphometric measurements, analyzed and interpreted the data, provided technical guidance for all aspects of the project, reviewed and edited the manuscript, and performed supervision. Nour Kahil performed histological procedures, hematological and biochemical procedures, performed the animal dosing and handling, collected, analyzed, and interpreted the data, performed a literature search, and original manuscript drafting. Najla Fakhruddin interpreted and analyzed the pathological results. Ramadan Awad synthesized and characterized the nanoparticles. Mahmoud I. Khalil validated and designed the study, reviewed and edited the manuscript, and was the principal investigator. All authors read and approved the final manuscript.

### Funding

The authors declare that no funds, grants, or other support were received for conducting this study.

### Data availability

The dataset presented in this study is available from the corresponding author upon reasonable request. 

### Compliance with ethical standards

All procedures involving animals were conducted and approved according to the guidelines of the Institutional Review Board (IRB) of Beirut Arab University, Beirut-Lebanon with a code number (IRB 2023-A-0049-S-M-0512). All laboratory animals received humanely care in compliance with the guidelines of the National Institutes of Health (NIH).

### Consent for publication

Not applicable.

### Conflict of interest

The authors declare that they have no competing interests or personal relationships that are relevant to the content of this article.

## Supplementary Material

Supplementary data

## Figures and Tables

**Table 1:**
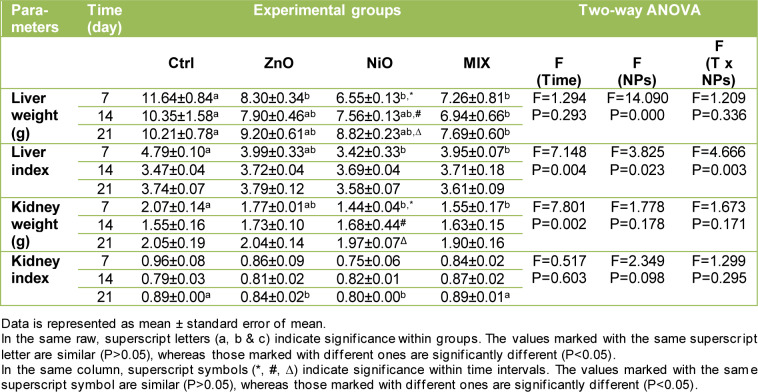
Changes in organ weight and indices between groups, over time intervals

**Table 2:**
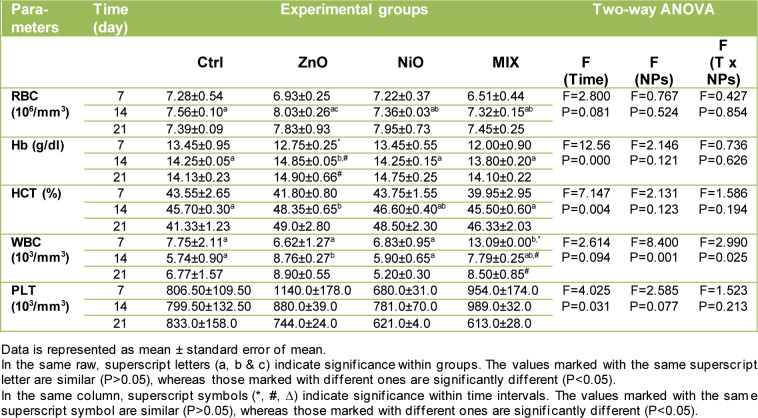
Changes in hematological parameters between groups, over time intervals

**Table 3:**
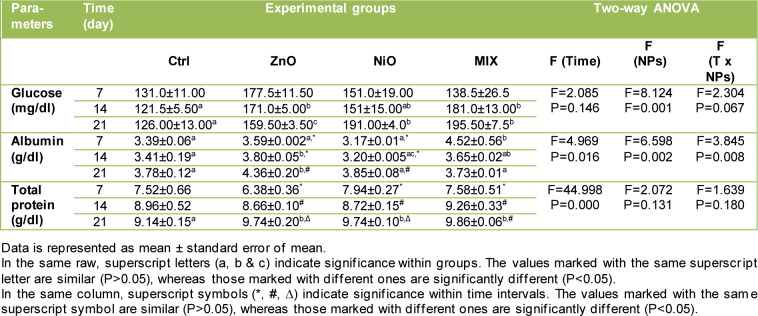
Changes in glucose, albumin and total protein content between groups, over time intervals
